# Eye movement desensitization and reprocessing therapy versus supportive therapy in affective relapse prevention in bipolar patients with a history of trauma: study protocol for a randomized controlled trial

**DOI:** 10.1186/s13063-017-1910-y

**Published:** 2017-04-04

**Authors:** Ana Moreno-Alcázar, Joaquim Radua, Ramon Landín-Romero, Laura Blanco, Mercè Madre, Maria Reinares, Mercè Comes, Esther Jiménez, Jose Manuel Crespo, Eduard Vieta, Victor Pérez, Patricia Novo, Marta Doñate, Romina Cortizo, Alicia Valiente-Gómez, Walter Lupo, Peter J. McKenna, Edith Pomarol-Clotet, Benedikt L. Amann

**Affiliations:** 1 FIDMAG Germanes Hospitalàries Research Foundation, Dr. Antoni Pujadas 38, 08830 Sant Boi de Llobregat, Spain; 2 Centre Forum Research Unit, Institute of Neuropsychiatry and Addictions (INAD) Hospital del Mar, Barcelona, Spain; 3 IMIM (Institut Hospital del Mar d’Investigacions Mèdiques), Barcelona, Spain; 4 Department of Psychiatry, Autonomous University Barcelona, Cerdanyola, Spain; 5 Division of Psychosis Studies, Institute of Psychiatry, Psychology and Neuroscience, King’s College London, London, UK; 6 Centre for Psychiatric Research and Education, Department of Clinical Neuroscience, Karolinska Institutet, Stockholm, Sweden; 7 Neuroscience Research Australia, Sydney, NSW Australia; 8 Macquairie University, Sydney, NSW Australia; 9 School of Medical Sciences, the University of New South Wales, Sydney, NSW Australia; 10 Benito Menni Complex Assistencial en Salut Mental, Sant Boi de Llobregat, Spain; 11 Hospital Clinic, Institute of Neuroscience, University of Barcelona, IDIBAPS, CIBERSAM, Barcelona, Catalonia Spain; 12 CIBERSAM (Centro de Investigación Biomédica en Red de Salud Mental), Madrid, Spain; 13 Bellvitge University Hospital, Psychiatry Department, Bellvitge Biomedical Research Institute (IDIBELL), Neurosciences Group-Psychiatry and Mental Health, Barcelona, Spain; 14 Clínica Logos, Barcelona, Spain

**Keywords:** Bipolar disorder, EMDR therapy, Supportive therapy, Psychological trauma, Treatment

## Abstract

**Background:**

Up to 60% of patients with bipolar disorder (BD) have a history of traumatic events, which is associated with greater episode severity, higher risk of comorbidity and higher relapse rates. Trauma-focused treatment strategies for BD are thus necessary but studies are currently scarce. The aim of this study is to examine whether Eye Movement Desensitization and Reprocessing (EMDR) therapy focusing on adherence, insight, de-idealisation of manic symptoms, prodromal symptoms and mood stabilization can reduce episode severity and relapse rates and increase cognitive performance and functioning in patients with BD.

**Methods/design:**

This is a single-blind, randomized controlled, multicentre trial in which 82 patients with BD and a history of traumatic events will be recruited and randomly allocated to one of two treatment arms: EMDR therapy or supportive therapy. Patients in both groups will receive 20 psychotherapeutic sessions, 60 min each, during 6 months. The primary outcome is a reduction of affective episodes after 12 and 24 months in favour of the EMDR group. As secondary outcome we postulate a greater reduction in affective symptoms in the EMDR group (as measured by the Bipolar Depression Rating Scale, the Young Mania Rating Scale and the Clinical Global Impression Scale modified for BD), and a better performance in cognitive state, social cognition and functioning (as measured by the Screen for Cognitive Impairment in Psychiatry, The Mayer-Salovey-Caruso Emotional Intelligence Test and the Functioning Assessment Short Test, respectively). Traumatic events will be evaluated by The Holmes-Rahe Life Stress Inventory, the Clinician-administered PTSD Scale and the Impact of Event Scale.

**Discussion:**

The results of this study will provide evidence whether a specific EMDR protocol for patients with BD is effective in reducing affective episodes, affective symptoms and functional, cognitive and trauma symptoms.

**Trial registration:**

The trial is registered at ClinicalTrials.gov, identifier: NCT02634372. Registered on 3 December 2015.

**Electronic supplementary material:**

The online version of this article (doi:10.1186/s13063-017-1910-y) contains supplementary material, which is available to authorized users.

## Background

Bipolar disorder (BD) is a serious neuropsychiatric condition of major public health importance with a complex multifactorial etiology, involving both genetic and environmental factors and associated with high mortality by suicide. BD is characterized by chronic instability of mood, circadian rhythm disturbances and continual fluctuations in energy levels, affect, sleep and cognitive appraisal of the self and others [1]. Although the disorder is conventionally characterized as being episodic in nature, with full recovery between periods of mania and depression, recent data suggest that many bipolar patients do not obtain a complete remission but continue to experience subsyndromal manic or depressive symptoms [2]. This is a clinically important issue since (1) subsyndromal symptoms are associated with a higher risk of relapses and poorer social functioning [3] and (2) interepisode affective instability seems to be refractory to pharmacological treatment [4]. Further possible risk factors for relapses include substance abuse, comorbid anxiety disorders, personality disorders or physical diseases [5].

The intervention strategies available for BD include essentially pharmacotherapy but also, increasingly, psychosocial interventions such as cognitive behavioral therapy, psychoeducation, interpersonal and social rhythm therapy, and family intervention [6]. However, despite the everyday use in clinical practice of both types of intervention, almost 70% of BD patients suffer from at least one affective relapse within 2 to 4 years [7–9].

The comorbidity of posttraumatic stress disorder (PTSD) with BD, with a prevalence of 16 to 39% of bipolar patients [10], might hereby have an important clinical implication. Traumatized bipolar patients suffer from more rapid cycling, more suicide attempts, more substance abuse, have a lower quality of life and more (hypo)manic and depressive symptoms than bipolar patients without PTSD [11, 12]. It is also of concern that early childhood adverse events, not necessarily fulfilling PTDS criteria, can trigger severe mental disorders – such as BD – later in life, with a negative impact on the course of the illness (e.g., [13]). Also, it is well established that adverse life events that occur later in life can negatively affect the course of the disease and can be important contributors to a poorer outcome (e.g., [14]). Studies hereby provide evidence that patients with severe mental illness and traumatic life events show more psychiatric symptoms, increased risk of suicide, increased frequency of risky sexual behaviors, more admissions to psychiatric hospitals and, overall, a greater risk of being re-traumatized [15, 16]. A recent naturalistic study over 4 years found an increase of depressive episodes in BD when patients were experiencing life events 6 months prior to the affective index episode [17].

However, this diagnostic comorbidity with significant negative effects on the prognosis of BD has been until recently largely ignored with an intriguing lack of trials for traumatized bipolar patients.

One form of treatment that is being increasingly used in PTSD is Eye Movement Desensitization and Reprocessing (EMDR) [18]. This integrative psychotherapeutic approach uses standardized protocols and elements of cognitive behavioral, interpersonal, and body-centered therapies in conjunction with dual stimulation (e.g., horizontal eye movements from side to side) [18, 19]. The results of two independent meta-analyses and reviews have shown that EMDR therapy is as effective in the treatment of PTSD symptoms as cognitive behavioral therapy [20–22]. The treatment with EMDR has also recently shown a beneficial and comparable effect to exposure therapy on patients with psychosis and comorbid PTSD in a large randomized controlled trial (RCT) [23]. Our group carried out the first pilot RCT of EMDR in bipolar patients with a history of traumatic events and subsyndromal symptomatology. Results showed that the EMDR intervention not only reduced the symptoms associated with trauma in the patients, but also had beneficial effects on subsyndromal affective symptoms [24]. Following these promising results, our research group has developed a specific and comprehensive EMDR protocol for bipolar patients with a history of trauma [25]. This protocol consists of a detailed survey of traumatic events, the intervention and processing of these events according to the Shapiro standard protocol [19] and five subprotocols directed to (1) mood stabilization, (2) enhancement of treatment adherence, (3) increase insight, (4) treatment of prodromal symptoms and (5) de-idealization of manic symptoms.

We hypothesized that our EMDR Bipolar protocol, as an adjunct to pharmacological treatment would have beneficial effects on the course of the illness of bipolar patients with a history of traumatic events.

## Methods/design

This multicenter collaborative project will involve the participation of three different centers from the Barcelona catchment area, Spain (Hospital Benito Menni, Hospital Clinic of Barcelona and Hospital del Mar). EMDR and supportive therapy (ST) therapists have extensive experience in the application of both psychotherapies. EMDR therapists have already participated in the pilot study [24] and all therapists received a detailed day training in applying the EMDR therapy protocol and a well-defined ST program. The application of all clinical assessments has been defined and practiced within all participating raters. All centers and research groups involved have wide ambulatory facilities and outpatient clinics for BD patients that will facilitate meeting the recruitment criteria and development of this project.

### Study aim

The overall aim of this study is to examine whether the EMDR Bipolar protocol for BP with a history of traumatic events can reduce affective relapses, improve affective and trauma-related symptoms and result in a better performance in cognition and psychosocial functioning, when compared ST. Other related aims of this trial are to expand the available options for psychosocial intervention in BD, to explore that the EMDR therapy is a safe and effective tool in traumatized bipolar patients and that treatment with EMDR leads to an improvement in the course and prognosis of the disease.

Based on our pilot study of EMDR in traumatized bipolar patients, we propose the following hypotheses:

1. The EMDR group will show fewer affective relapses at 12 and 24 months’ follow-up compared to the ST group
2. Patients in the EMDR group will show fewer affective and trauma-related symptoms compared to the ST group at evaluations after 6 months of therapy, at 12 and 24 months
3. Patients in the EMDR group will show a better cognitive and psychosocial functioning compared to the ST group at evaluations after 6 months of therapy, at 12 and 24 months

### Trial design

**Fig. 1:**
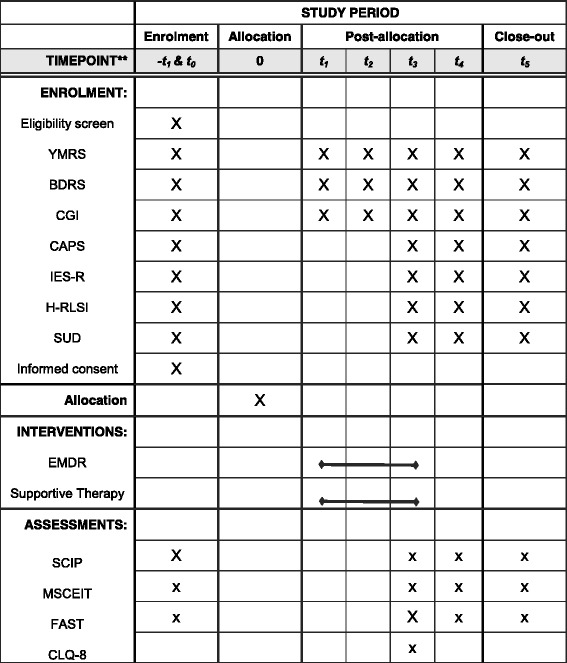
Flow diagram of enrollment, intervention and assessments. YMRS: Young Mania Rating Scale; BDRS: Bipolar Depression Rating Scale; CGI-BP-M: Clinical Global Impression, PTSD: Posttraumatic Stress Disorder; CAPS: Clinician Administered PTSD Scale; IES-R: Impact Event Scale; H-RLSI: The Holmes-Rahe Life Stress Inventory; SUD: Subjective Units of Distress; SCIP: Screen for Cognitive Impairment in Psychiatry; MSCEIT: The Mayer-Salovey-Caruso Emotional Intelligence Test; FAST:Functioning Assessment Short Test; CLQ-8: Client Satisfaction Questionnaire

This is a single-blind, randomized controlled clinical trial with two parallel branches, EMDR and ST. Patients will be matched for age, sex, illness duration and number of affective episodes the year before the clinical trial. The primary outcome variables that are affective relapses, symptoms of trauma and cognition and psychosocial functioning, will be assessed at six time points; pretreatment (T0), mid-treatment at 2 weeks (T1) and at 3 months (T2), post treatment at 6 months (T3), and follow-up evaluations at 12 (T4) and 24 months (T5) (see Fig. 1). The secondary outcome variables that are the course and prognosis of the disease, will be assessed in follow-up evaluations at 12 (T4) and 24 months (T5). The research assistants conducting the clinical assessments will be blind to the participants’ research condition. Patients are not blind to treatment as it is impossible for a therapist to offer and for a patient to receive therapy without being aware of the techniques that they are using orreceiving. The study has been approved by the Ethics Committee of the Germanes Hospitalàries del Sagrat Cor de Jesús (reference number: PR-2014-15), the Hospital Clínic of Barcelona (reference number: HCB/2015/1005) and the Hospital del Mar (reference number: 2015/6502/l). All participants will sign informed consent prior to enrollment. Details of the trial design can be also gathered from Additional file 1 (Standard Protocol Items: Recommendations for Interventional Trials (SPIRIT) flow diagram) and the SPIRIT Checklist (Additional file 2).

### Participants

The patient sample will consist of 82 patients fulfilling *Diagnostic and Statistical Manual of Mental Disorders, fourth edition, text revision* (DSM-IV-TR) criteria for bipolar I and II disorder, based on clinical interview and review of case notes. Inclusion criteria will be: (1) age between 18 and 65 years, (2) presence of between two and six affective episodes in the last year, (3) current clinical status of euthymia upon clinical evaluation, defined as the presence of clinical remission (Bipolar Depression Rating Scale (BDRS) <8 and Young Mania Rating Scale (YMRS) <6; or subsyndromal status defined as BDRS ≥8 and <14 and/or YMRS ≥7 and <12), (4) presence of one or more traumatic events causing current trauma-related symptoms (The Holmes-Rahe Life Stress Inventory, H-RLSI >0; The Clinician-administered PTSD Scale (CAPS): frequency event >1, intensity of symptom >2; Impact of Event Scale-Revised, IES-R >0), and – following the standard EMDR protocol [19] – Subjective Units of Distress (SUD) >5 (range from 0 to 10, with a score of 10 meaning maximum stress).

Exclusion criteria will be: (1) substance abuse/dependency within 3 months prior to participation (except of nicotine abuse/dependency), (2) neurological disease or brain trauma history, (3) suicidal ideation, (4) previous involvement in trauma-focused therapy, (5) current psychotherapy during the study and months of follow-up.

### Randomization procedure

All patients meeting the inclusion criteria receive the pretreatment (T0) assessment. After T0, participants will be assigned to EMDR or the ST group by two independent researchers using the following covariate-adaptive allocation procedure [26]: (1) the first two patients will be randomly allocated to EMDR or ST with *p* = 0.5, (2) the next patients will be allocated as follows: (b1) if one group already includes two more patients than the other group, the patient will be randomly allocate to EMDR or ST with *p* = 0.8 for the smallest group and *p* = 0.2 for the largest group, (b2) otherwise, we will first simulate that the patient is allocated to EMDR and calculate the sum of the between-group square standardized differences in age, sex, illness duration and number of affective episodes the year before the clinical trial between groups, we will then simulate that the patient is allocated to ST and recalculate the sum, and finally randomly allocate the patient to EMDR or ST with *p* = 0.8 for the group with the smallest sum and *p* = 0.2 for the group with the largest sum. For example, if we had already included 10 patients to the EMDR group and 8 patients to the ST group, the 19th patient would be randomly allocated with *p* = 0.2 for EMDR and *p* = 0.8 for ST. If they were allocated to ST, we would calculate the above sum of covariates after simulating that the 20th patient is allocated to EMDR and after simulating that is allocated to ST, and if the sum of the EMDR simulation was larger than the sum of the ST simulation, we would randomly allocate the 20th patient with *p* = 0.2 for EMDR and *p* = 0.8 for ST. Following this procedure, final groups should be balanced in size and matched in age, sex, duration and episodes. All steps of the randomization process will be automatically carried out in a central location using a computer program.

### Computation of sample size

The main aim of the study is to assess the relative efficacy of a specific EMDR intervention protocol versus ST therapy in the clinical stabilization (decrease in the risk of relapse) of traumatized bipolar patients. For this reason the reduction of the risk of relapse after treatment with a follow-up of 24 months will be used as the dependent variable of the study. The calculation of the sample size was based on a survival analysis using the statistical package “powerSurvEpi” for R (http://www.r-project.org/) using an alpha = 0.005 instead of 0.05 to allow correction for multiple comparisons. The number of patients required to detect a hazard ratio = 2 (i.e., the hazard rate of relapse is one group is the double of the hazard rate in the other group) in a Cox regression with a statistical power of 80% and alpha = 0.005 is *n* = 36 per intervention group (two groups, EMDR and ST, equals to a total *n* = 72). According to Chambless and Hollon (1998) a sample of this size should show clinically relevant differences [27]. Assuming a percentage of dropouts of about 10–15% of the participants it would be necessary to recruit approximately 82 patients, 41 allocated in each intervention arm.

### Interventions

#### Eye Movement Desensitization and Reprocessing (EMDR)

EMDR is a psychotherapeutic approach designed to alleviate the distress associated with traumatic memories facilitating the access and process of traumatic memories and other adverse life experience to bring these to an adaptive resolution. The EMDR therapy will consist of 20 individual sessions 60 min each over a period of 6 months. As stated before, the EMDR Bipolar protocol will include an evaluation of the psychiatric history of the patient and an assessment of dysfunctional cognitions and identification of targets in the context of traumatic events. In addition, the reprocessing of traumatic events will follow the standardized protocol of Shapiro [19] together with the five subprotocols specific for BD that target the following areas:

1. *Mood stabilization*: the major goal in the treatment process of bipolar patients is the long-term normalization of mood. The aim of this protocol is to reinforce the positive experiences of affective stability and self-control in bipolar patients. Via bilateral stimulations this protocol uses also positive cognitions to strengthen the adaptive system of beliefs. When this installation is reinforced repeatedly, patients are expected to experience an improvement in their self-esteem and affective stability that, in turn, is connected with positive body sensations
2. *Treatment adherence*: poor adherence in bipolar patients is mainly caused by the feeling of being controlled by drugs, (hypo)manic episodes, lack of insight, a negative view on pharmacological treatment, substance abuse, lack of treatment response and side effects such as weight gain and sedation [27]. This protocol aims to identify and improve those issues and strengthen adherence to avoid further affective relapses. First, we find out what subjective problems are connected to the psychopharmacological treatment. Then, the therapist suggests various positive consequences from taking medication. These are then connected to positive cognitions, emotions or body sensations, and installed via bilateral stimulation
3. *Illness awareness*: the aim is to help clients be more aware of their disease. Good awareness is associated with fewer affective symptoms and risky behavior, better adherence to treatment, and a healthier lifestyle. Being aware of BD can help the client to take medication more reliably, act more consistently concerning eating and sleeping habits, reduce risky behaviors, and facilitates asking for professional help when necessary. In this protocol, the therapist reviews with the patient which kind of affective symptoms suggestive of BD experienced the subject in the past. Then, the therapist proposes positive beliefs connected to the fact of having experienced symptoms and being aware of them; this will be reinforced with bilateral stimulation
4. *Detection of prodromal symptoms*: the aim of this protocol is to help bipolar patients to identify early prodromal symptoms such as sleep disturbances, mood changes/lability, psychotic symptoms, agitation, restlessness, increased anxiety and changes in appetite or suicidal ideas. In this way, patients can request a rapid therapeutic intervention to avoid an affective relapse. After the identification of individual prodromal symptoms, the therapist proposes a list of positive beliefs connected to the ability to identify prodromal symptoms. Then, the therapist reinforces prodromal symptom awareness with positive cognitions using bilateral stimulation
5. *De-idealization of pleasurable manic symptoms*: the aim of this protocol is to assist patients who are aware of manic episodes but still idealize specific pleasant euphoric symptoms and ignore the devastating consequences of these symptoms during a manic episode, running a higher risk of poor adherence and further affective relapses. First, the therapist creates with the patient a list of manic-state life experiences that ended in disastrous consequences and connect each experience that occurs during a mixed or manic state with the corresponding impulse, action, sensation, belief and thought. Then, the therapist strengthens via bilateral stimulation the patient’s awareness connecting manic symptoms and impulsivity with disastrous consequences

Some EMDR sessions will be recorded on video randomly and will be evaluated by an external expert in EMDR to verify that the protocol is being administered properly.

#### Individual supportive therapy (ST)

ST is a psychological treatment that encourages patients to express and evaluate their life situation. Living with a mental disease can significantly impact all major domains of life and this can understandably provoke in patients negative feelings such as frustration, helplessness or depressive symptoms. ST also consists of 20 individual sessions of 60 min each over a period of 6 months. One of the main objectives of ST is to offer an opportunity for the subjects to express how their lives are affected by the illness. The ST is a patient-centered, active-listening intervention that enables the patient to receive emotional support and counselling. We also included general advice and information about BD and medication without referring to written or structured material. We offer an unstructured training to enhance assertive behavior; we also include relaxation exercises, problem-solving training and the use of a mood diary or specific mobile app as a tool for tracking the warning signs of a bipolar episode. Single ST was chosen as control condition because it provides an intervention for the patient of similar intensity but with no particular focus on the processing of traumatic events or on structured psychoeducative interventions of the disorder.

### Dropouts and follow-up

In case of an affective relapse with admission to the hospital during the intervention phase of 6 months patients will be excluded from the trial and considered as dropouts. In case of relapse which requires an admission to the hospital during the follow-up, patients will be maintained in the trial to obtain maximum information on the course of the illness.

### Assessments

Demographical and clinical variables will be collected through an interview using the medical history of the patients and a specific Case Report Form (CRF) for the study, including sex, age, age of onset of the disorder, number of relapses, number of previous episodes, history and number of suicide attempts previous and current pharmacological treatment, family background and consumption of drugs. To ensure privacy of participants and safe data storage, data will also be entered in a data file anonymously; participants will be given a unique number, and only the research assistant will be able to link the number with the name of the patient. At baseline (T0) research assistants will also assess clinical severity of affective symptoms, presence of traumatic live events and trauma symptoms, and cognitive and psychosocial functioning. At 2 weeks (T1) and 3 months (T2) of treatment patients will be evaluated with respect to clinical symptoms. After the treatment phase (T3) subjects will be interviewed with respect to clinical symptoms, trauma scales, cognitive and psychosocial functioning and patient satisfaction. During two follow-up visits at 1 year (T4) and 2 years (T5), blind-to-treatment evaluators apply clinical affective and trauma scales, functioning and cognition. See Fig. 1 (flow diagram of enrollment, intervention and assessments).

The clinical severity will be assessed by means of different instruments (see Table 1):

**Table 1:** Measurements to evaluate clinical variables

Clinical variable	Measurement interview/Self-report	T0	T1	T2	T3	T4	T5
		Baseline	Mid-treatment		Post treatment	FU 12 months	FU 24 months
Mania	Young	x	x	x	X	x	x
Depression	BDRS	x	x	x	X	x	x
Severity	CGI-BP-M	x	x	x	X	x	x

*FU* Follow-up, *YMRS* Young Mania Rating Scale, *BDRS* Bipolar Depression Rating Scale, *CGI-BP-M* Clinical Global Impression Scale modified for bipolar disorder

1. The BDRS [28], Spanish validation (BDRS-S) [29]: the BDRS is the most up-to-date and appropriate tool to assess depressive and mixed symptoms in DSM-IV-diagnosed bipolar I and II patients. The BDRS comprises 20 items that evaluate the clinical features associated with the depressive phase of BD, including atypical symptoms and mixed phenomenology [28]. Symptom severity are rate on a 4-point scale ranging from 0 = no symptoms present, 1 = mild, 2 = moderate, 3 = severe
2. The YMRS [30], Spanish validation [31], is a heteroadministered scale composed of 11 items aimed at quantifying the severity of manic and hypomanic episodes. There are four items that are graded on a 0 to 8 scale (irritability, speech, thought content and disruptive/aggressive behavior), while the remaining seven items are graded on a 0 to 4 scale. These four items are given twice the weight of the others to compensate for poor cooperation from severely ill patients. Strengths of the YMRS include its brevity, widely accepted use, and ease of administration
3. The Clinical Global Impression Scale modified for BD (CGI-BP-M) [32], Spanish validation [33]: the CGI-BP-M scale consists of three subscales; the first two assess the severity of acute symptoms of mania and depression, and the third evaluates the longitudinal severity of the disease. Each subscale has seven subcategories with scores from 1 to 7 rating the severity of the disorder as normal, low, mild, moderate, marked, severe or very severe

Trauma symptoms will be evaluated using the tools listed in Table 2:

**Table 2:** Measurements to evaluate trauma symptoms

Clinical variable	Measurement interview/Self-report	T0	T1	T2	T3	T4	T5
		Baseline	Mid-treatment		Post treatment	FU 12 months	FU 24 months
PTSD	CAPS	x			x	x	x
Adverse events	IES-R	x			x	x	x
	H-RLSI	x					
	SUD	x			x	x	x

*FU* Follow-up, *PTSD* Posttraumatic Stress Disorder, *CAPS* The Clinician-administered PTSD Scale, *IES-R* Impact Event Scale, *H-RLSI* The Holmes-Rahe Life Stress Inventory, *SUD* Subjective Units of Distress

1. The Clinician-Administered PTSD Scale (CAPS) [34], Spanish validation [35]: the CAPS is the standard scale in the evaluation of the presence or absence of PTSD diagnosis and the frequency and intensity of the symptoms. This scale provides ratings of the frequency and intensity of each of the 17 DSM-IV-TR-based PTSD symptoms on a 0 to 4 Likert-type scale, thereby allowing for a maximal score of 8 for each symptom and a total score range from 0 to 136 [23]. It can be used to diagnose a current or past trauma, or to evaluate symptoms during the last week
2. The Impact of Event Scale Revised (IES-R) [36], Spanish validation [37]: the IES-R is a 22-item self-report measure that assesses subjective distress caused by traumatic events. Items correspond directly to 14 of the 17 DSM-IV symptoms of PTSD. Patients are asked to identify a specific stressful life event and then indicate how much they were distressed or bothered during the past 7 days. Items are rated on a 5-point scale ranging from 0 = not at all, 1 = a little bit, 2 = moderately, 3 = quite a bit and 4 = extremely. The IES-R yields a total score (ranging from 0 to 88) and subscale scores can also be calculated for the Intrusion, Avoidance and Hyperarousal subscales
3. The Holmes-Rahe Life Stress Inventory [38], Spanish validation [39]. This scale assesses frequency of 43 common stressful life events that occurred over the previous 12 months, providing a standardized measure of the impact of these stressors [40]. Interpretation of the overall score is difficult because of the large differences in each person’s ability to cope and their particular reactions to stress, but there are some general guidelines. Scores below 150 reflect low levels of stress, scores between 150 and 299 represent a 50% risk of illness in the near future and scores above 300 represent an 80% risk of developing a stress-related illness [38]

Cognitive profiles and overall functioning will be evaluated using (see Table 3):

**Table 3:** Measurements to evaluate cognitive and functional profiles and satisfaction with the treatment

Clinical variable	Measurement interview/Self-report	T0	T1	T2	T3	T4	T5
		Baseline	Mid-treatment		Post treatment	FU 12 months	FU 24 months
Cognition	SCIP	x			x	x	x
	MSCEIT	x			x	x	x
Functionality	FAST	x			x	x	x
Satisfaction	CLQ-8				x		

*FU* Follow-up, *SCIP* Screen for Cognitive Impairment in Psychiatry, *MSCEIT* The Mayer-Salovey-Caruso Emotional Intelligence Test, *FAST* Functioning Assessment Short Test, *CLQ-8* Client Satisfaction Questionnaire

1. Screen for Cognitive Impairment in Psychiatry (SCIP) [41], Spanish validation [42]: the SCIP is a short tool to assess cognitive impairment in psychiatric patients and consists of five subtests that assess immediate and delayed verbal learning, working memory, verbal language, and processing speed. Three alternate forms facilitate their application to assess possible cognitive changes and avoid habituation and learning effects in repetitive within-patient evaluations. The SCIP-S yields a total score and subscale scores can also be calculated for every single cognitive function evaluated
2. The Mayer-Salovey-Caruso Emotional Intelligence Test (MSCEIT) [43], Spanish validation [44]. The MSCEIT evaluates emotional intelligence based on scenarios typical of everyday life. This test measures how well people perform tasks and solve emotional problems, rather than having them provide their own subjective assessment of their emotional skills [43]. The MSCEIT consists of 141 items and the results are presented in a comprehensive personal summary report providing graphical representations of 15 separate emotional intelligence scores and a detailed explanation of score meanings. The MSCEIT provides a total score, two general areas of emotional intelligence (experiential and strategic), four branches of emotional intelligence (perceiving emotions, using emotions, understanding emotions and managing emotions) and eight specific emotional intelligence tasks (identifying emotions in pictures and in faces, relating emotions to sensation and to thinking, analyzing complex emotions and chains of emotions, and incorporating into decision-making your own emotions and the emotions of others)
3. Functioning Assessment Short Test (FAST) [45]: the FAST is a brief instrument to evaluate performance in six different areas of functioning, such as autonomy, occupational functioning, cognitive functioning, finances, relationships and leisure. This test contains 24 items and all of them are rated on a 4-point scale. The global score is obtained by summing the scores of each item (ranging from 0 to 72); higher scores indicate poorer functional status

Finally, to measure patient satisfaction with the treatment, we will use the Client Satisfaction Questionnaire (CSQ-8) [46], Spanish validation [47]. The CSQ-8 is an eight-item, easily scored and administered measurement that is designed to measure client satisfaction with services. The items for the CSQ-8 were selected on the basis of ratings by mental health professionals of a number of items that could be related to client satisfaction and by subsequent factor analysis. The CSQ-8 is unidimensional, yielding a homogeneous estimate of general satisfaction with services. The CSQ-8 provides a total score (ranging from 8 to 32), with high scores indicating greater satisfaction.

## Statistical analysis

The baseline distribution of sociodemographic and clinical characteristics in each treatment arm will be reported using descriptive statistics.

The main hypothesis of the change in the time to relapse (in weeks) will be analyzed using Cox regressions, and the hypotheses of the changes in the scores of clinical scales as compared to baseline will be tested using repeated measures analyses of variance (ANOVAs) that will include time, treatment arm and their interaction. Those variables that show statistically significant between-arm differences at baseline will be added as covariates in the Cox regressions and repeated measures ANOVAs. For each of the analyses effect size measures (hazard ratio or Hedges’ *g*) will be estimated.

An intention-to-treat (ITT) analysis will be employed to provide unbiased comparisons among the treatment groups, using multiple imputations to deal with the losses of follow-up.

R software will be used to carry out the statistical analyses.

## Discussion

This trial is based on our first preliminary RCT of EMDR in traumatized bipolar patients, with positive results in affective and trauma-related symptoms in the EMDR group when compared to treatment as usual [24]. The main goal of the current study is to test in a large multicenter sample of bipolar I and II patients, if a newly developed, specific EMDR protocol for BD [25], compared to ST, can reduce affective relapses, improve affective and trauma-related symptoms and result in a better performance of cognition and functioning. We believe that this trial is an important contribution in the treatment of bipolar patients because it aims to improve the course and prognosis of the disorder and, therefore, their quality of life and overall functionality. As one of the increasingly used psychotherapeutic tools [21], we want to explore whether or not EMDR may represent a potentially relevant therapeutic strategy for bipolar I and II patients with traumatic events in their history. Together with the work on trauma-related symptoms, we will target four specific risk factors for relapses such as lack of insight, bad adherence, insufficient awareness of prodromal symptoms and de-idealisation of manic symptoms. The fifth subprotocol consists of an EMDR intervention directed towards mood stabilization via bilateral stimulation. But why do we believe that EMDR might have a mood-stabilizing effect on bipolar patients? This idea is based on a recent study which suggested that EMDR might modulate the Default Mode Network (DMN) in traumatized bipolar patients [48]. First identified in 2001, the DMN is an interconnected series of brain regions, including prominently the medial frontal cortex and also the posterior cingulate cortex/precuneus, which are highly active at rest but deactivate during the performance of attention-demanding tasks [49, 50]. DMN dysfunction is currently implicated in major psychiatric disorders, such in BD across all phases of the disorder [51]. Interestingly, another study has also found alterations in the DMN in PTSD [52]. In our study [48] we found marked improvement after receiving EMDR for subsyndromal mood symptoms. Surprisingly, the patient also showed changes on functional magnetic resonance imaging (MRI) in the direction of normalization of the DMN. This suggests that some of the persisting neurofunctional changes which have been found to characterize BD may not in fact be immutable but can change alongside with changes in clinical status. This preliminary result needs replication but provides a possible neurobiological rational behind the mechanism of action of EMDR.

To understand better the relationship between the presence of traumatic events and BD, we will study several variables before, during and after treatment. We will assess clinical severity, cognitive and overall functioning variables with specific and validated instruments.

This study aims to replicate the positive results of our pilot study in a larger sample of BD patients, thus strengthening the role of EMDR as a therapy and providing a fast and safe additional tool to established psychosocial interventions that are currently available in the treatment of BD. Strengths of this trial include an innovative, relatively new psychotherapeutic tool (which fills a striking lack of data in the field), a solid methodology and a large observational period of 2 years. The results of this trial are expected to spawn several publications in high-impact factor journals and, if the treatment is useful, will be integrated in future clinical treatment guidelines for BD.

## Limitations

Differences in pharmacological treatment received by patients could be a possible source of clinical noise. To partly overcome this limitation, the “pharmacological treatment” variable will be taken into account in the statistical analysis. Moreover, it should be noted that all participating centers have extensive experience in the treatment of BD and use standardized treatment protocols, which would also contribute to limit this potential issue.

## Trial status

Intervention and assessment training was performed in June 2016 and patient recruitment began in July 2016.

## Additional files

Additional file 1: SPIRIT flow diagram. (PDF 83 kb)

Additional file 2: SPIRIT Checklist. (DOC 122 kb)

## Abbreviations

BDRS: Bipolar Depression Rating Scale
CAPS: The Clinician-Administered PTSD Scale
CGI-BP-M: Clinical Global Impression Scale modified for bipolar disorder
CRF: Case Report Forms
CSQ: Client Satisfaction Questionnaire
EMDR: Eye Movement Desensitization and Reprocessing
FAST: Functioning Assessment Short Test
IES-R: Impact of Event Scale-Revised
ITT: Intention-to-treat
MSCEIT: The Mayer-Salovey-Caruso Emotional Intelligence Test
PTSD: Posttraumatic stress disorder
SCIP: Screen for Cognitive Impairment in Psychiatry
ST: Supportive therapy
SUD: Subjective Units of Distress
YMRS: Young Mania Rating Scale
