# Supplementary figures and images for: Eye movement desensitization and reprocessing therapy versus supportive therapy in affective relapse prevention in bipolar patients with a history of trauma: study protocol for a randomized controlled trial
Source: Trials. 2017 Apr 4;18:160. doi: 10.1186/s13063-017-1910-y (PMC5379519; doi:10.1186/s13063-017-1910-y)

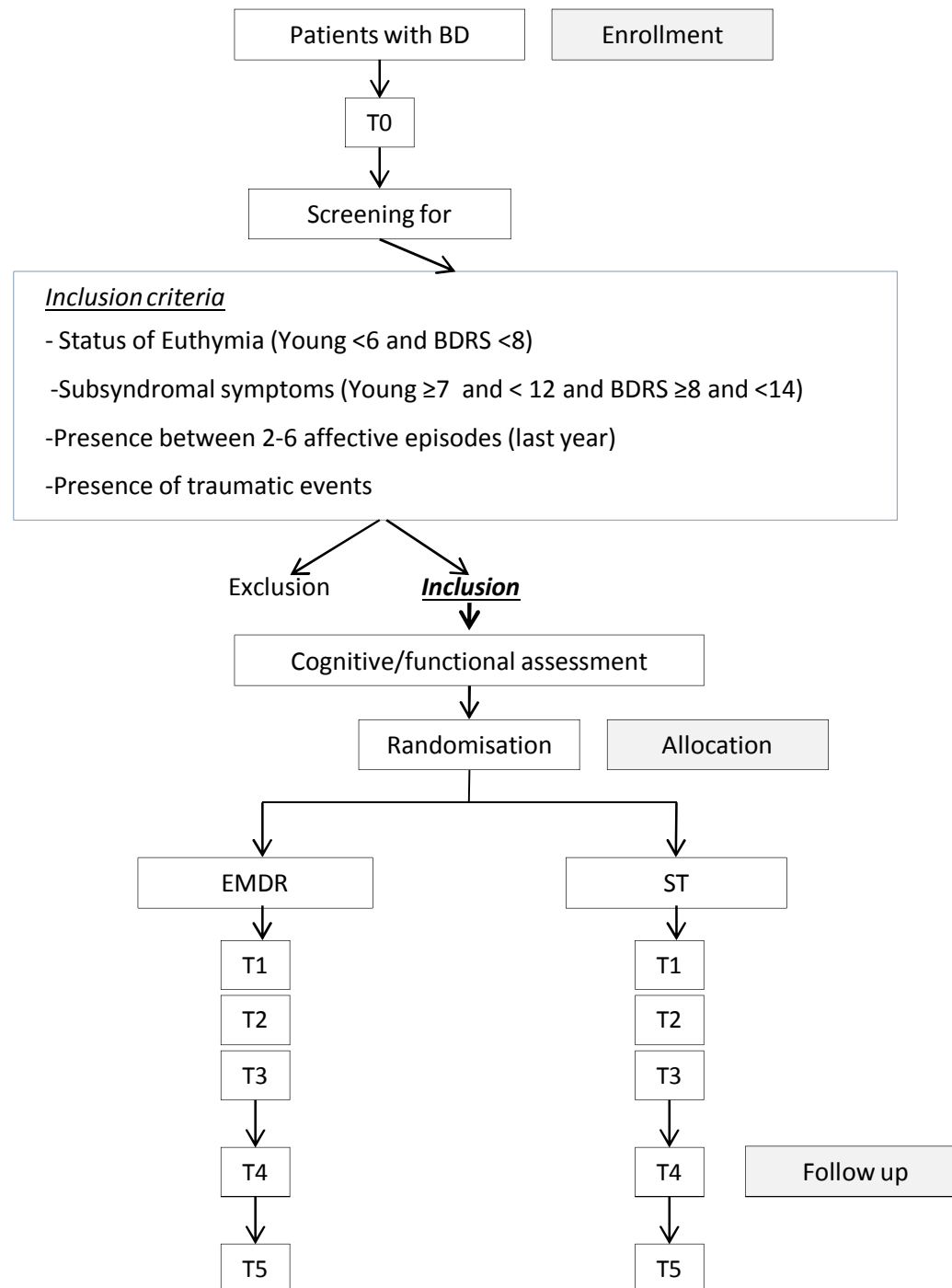

Supplement: Supplementary file 1 — SPIRIT flow diagram. (PDF 83 kb) [file 13063_2017_1910_MOESM1_ESM.pdf]
